# High-resolution app data reveal sustained increases in recreational fishing effort in Europe during and after COVID-19 lockdowns

**DOI:** 10.1098/rsos.230408

**Published:** 2023-07-19

**Authors:** Asta Audzijonyte, Fernando Mateos-González, Justas Dainys, Casper Gundelund, Christian Skov, J. Tyrell DeWeber, Paul Venturelli, Vincentas Vienožinskis, Carl Smith

**Affiliations:** ^1^ Institute for Marine and Antarctic Studies, University of Tasmania, Tasmania, Australia; ^2^ Nature Research Centre, Akademijos 2, Vilnius, Lithuania; ^3^ Centre for Marine Socioecology, Tasmania, Australia; ^4^ ALKA Wildlife, Lidéřovice, Czech Republic; ^5^ Section of Freshwater Fisheries and Ecology, Technical University of Denmark, Denmark; ^6^ Potsdam Institute of Inland Fisheries, Im Königswald 2, Potsdam, Germany; ^7^ Department of Biology, Ball State University, Muncie 47306, IN, USA; ^8^ Deeper, LT-10312 Vilnius, Lithuania; ^9^ Department of Ecology and Vertebrate Zoology, University of Łódź, Łódź, Poland; ^10^ Institute of Vertebrate Biology of the Czech Academy of Sciences, Brno, Czech Republic

**Keywords:** anthropause, recreational fishing effort, non-probabilistic methods, smartphone applications, COVID-19, inland and coastal fisheries

## Abstract

It is well recognized that COVID-19 lockdowns impacted human interactions with natural ecosystems. One example is recreational fishing, which, in developed countries, involves approximately 10% of people. Fishing licence sales and observations at angling locations suggest that recreational fishing effort increased substantially during lockdowns. However, the extent and duration of this increase remain largely unknown. We used four years (2018–2021) of high-resolution data from a personal fish-finder device to explore the impact of COVID-19 lockdowns on angling effort in four European countries. We show that relative device use and angling effort increased 1.2–3.8-fold during March–May 2020 and generally remained elevated even at the end of 2021. Fishing during the first lockdown also became more frequent on weekdays. Statistical models explained 50–70% of the variation, suggesting that device use and angling effort were relatively consistent and predictable through space and time. Our study demonstrates that recreational fishing behaviour can change substantially and rapidly in response to societal shifts, with profound ecological, human well-being and economic implications. We also show the potential of angler devices and smartphone applications for high-resolution fishing effort analysis and encourage more extensive science and industry collaborations to take advantage of this information.

## 1. Introduction

The COVID-19 pandemic and associated lockdowns affected the natural world and provided novel research opportunities. In many cases, the slowing of human activities, coined the ‘Anthropause’ [1], had positive effects on nature through reduced traffic [2,3], noise and other pollution [4,5], airspace fragmentation and human activity in coastal areas [6–8] (but see Bates *et al*. [9] for the full range of impacts). There is also evidence that the Anthropause had a positive effect on marine ecosystems and fishes [10,11] through reductions in small-scale [12–15] and, to a lesser degree, large-scale commercial fisheries [16]. For example, commercial seafood catches in the US alone were estimated to be 40% lower in 2020 compared with 2019 [17]. By contrast, the impacts of COVID-19 on coastal and freshwater ecosystems and recreational fishing are more uncertain [9,18]. Recreational fishing is a hugely important social, cultural, economic and ecological activity globally, especially in the developed world, where an estimated 10% of people participate [19,20]. Global estimates suggest that nearly 0.7 billion people engage in recreational fishing, which represents a substantial contribution to local economies [21], an essential source of protein [22] and important well-being and health benefits [23]. Recreational fishing also constitutes the major or sole source of fishing mortality in many freshwater and coastal fish stocks. It can substantially impact fish populations and entire ecosystems [24–26].

Despite the ecological and societal importance of recreational fishing, large-scale estimates of recreational fishing effort and potential impacts still need to be determined [27]. This uncertainty stems from a lack of temporally and spatially resolved data for recreational fishing effort, because many countries do not require anglers to report their fishing trips or catch. In most countries, recreational fishing effort is assessed via fishing licence sales, creel surveys, questionnaires or expert knowledge, all of which may not reflect actual fishing effort and changes in angling behaviour, especially in atypical circumstances, such as a pandemic. Nevertheless, existing evidence provides insights into recreational fishing behaviour during government-imposed COVID-19 lockdowns. For example, a survey of diary panellists in the UK suggested that the fishing effort of sea anglers declined during 2020 in response to the lockdown [28]. By contrast, the sale of angling licences in Denmark increased by 20% in March–May of 2020 [29], first-time or returning anglers increased by 20% in Ontario, Canada [21], fishing trips increased by 20% in the United States [30], and apparent illegal fishing increased approximately 10-fold in some marine-protected areas in British Columbia, Canada [31]. A more nuanced pattern was observed in Western Australia, where metropolitan boat anglers seemingly fished less due to travel restrictions, but anglers outside of urban centres increased the frequency of fishing trips [32]. In general, these studies suggest a modest (*ca* 20%) and temporary increase in recreational fishing effort and probably contrasting patterns of angler behaviour across countries that depended on country-specific regulations and traditions. For example, access to fishing was restricted for the British, whereas Danes were encouraged to spend time outdoors. However, many of these studies were limited to relatively small spatial or temporal scales and often focused only on the initial stages of lockdowns. As a result, these studies could not answer the questions about potential COVID-19-driven changes in angler behaviour with a fine temporal and spatial resolution across large regions or determine if behaviour changed in the long term following lockdowns.

Recreational fisheries are complex, adaptive social-ecological systems [27,33] potentially more sensitive to societal changes than commercial fisheries. Major changes in recreational fishing are likely to have important consequences for target fish species, local economies and angler health and well-being. The COVID-19 pandemic provides a significant opportunity to understand angler behaviour—not only in response to rare events like the Anthropause, but also as a natural experiment that can provide insights into more general aspects of human behaviour in the context of recreational fisheries management. Novel digital data, such as human mobility, social media or smartphone application data, have already provided unexpected insights into human behaviour changes in health, transportation, education and economic activity [34–38]. Given the extensive angler engagement with modern technology and apps [39,40], there is great potential to revolutionize recreational fisheries research by tapping into these novel data sources [41]. In this study, we attempt a large-scale assessment of angler behaviour changes using high-resolution anonymous personal sonar device data as a proxy for recreational angler fishing effort in four European countries with different socio-economic and urbanization levels and different recreational fishing management regimes or frameworks (Lithuania, Czech Republic, Denmark and Germany). We show how these data can be used to provide new and detailed knowledge on angling effort through space and time. We ask three main questions to understand how COVID-19 affected recreational fisher behaviour and, by inference, also their potential impacts on aquatic ecosystems. First, did angling effort change during COVID-19 lockdowns, and were there common patterns among countries? Second, if changes occurred, were they sustained once the lockdowns ended? Third, which factors explain the estimated angling effort through space and time, and can our statistical models explain this effort distribution?

## 2. Methods

### 2.1. Data

Recreational fishing effort in the Czech Republic, Denmark, Germany and Lithuania was assessed using anonymous data from a widely used low-cost portable fish finder device *Deeper Sonar* (https://deepersonar.com/). Recreational anglers use this device to locate fish, measure water depth and create small-scale bathymetric maps to aid angling. A recent study calibrated the proportion of anglers that used this sonar device at a popular fishing location in Lithuania during 2020–2021 [42] and showed high correspondence between the proportion of device users and the actual number of anglers on a given day (95% posterior probabilities of 1.5–2.6% of anglers during the ice-free period and higher proportion for ice fishing). These findings imply that angler behaviour and total recreational fishing effort may be reasonably well predicted from sonar usage. However, the quality of this prediction is likely to vary among countries (see Discussion). Therefore, we explored daily angler sonar usage counts per municipality, county or other administrative unit, as appropriate, in each of the four countries and assessed how this usage compared before, during and after COVID-19 lockdowns, using the lockdown stages and definitions provided by Our World in Data compilation (electronic supplementary material, figure S1). Depending on the country, the number of unique sonar users by the end of 2021 constituted 0.35–10% of the estimated total anglers (table 1) [43]. We assumed that changes in sonar device use reflected changes in the overall angling effort, but see Discussion for further clarifications and caveats.

**Table 1.** Summary data for the four countries included in the study, with lockdown phases taken from Our World in Data compilation of stay-at-home requirement in the ‘Policy Responses to the Coronavirus Pandemic’, and government data for Denmark (see electronic supplementary material). The total number of unique sonar users and fishing trips recorded is shown for the last day of the phase. The 2020 population of each country is shown in millions [43]. For details on angler number estimation, see Methods. Percentage shows the proportion of unique sonar device users at the end of the study period relative to the total estimated number of anglers.

country (population size in 2020)	lockdown phase	start	end	days	unique users	trips recorded	estimated angler numbers
Czech Republic (10.7 M)	pre-lockdown	01-01-2018	14-03-2020	804	969	15 437	320 K
	lockdown 1	15-03-2020	31-05-2020	77	3957	19 232	(approx. 2%)
	post-lockdown 1	01-06-2020	21-10-2020	142	4773	26 210	
	lockdown 2	22-10-2020	11-04-2021	171	5382	29 843	
	post-lockdown 2	12-04-2021	31-12-2021	264	6979	42 297	
Denmark (5.8 M)	pre-lockdown	01-01-2018	10-03-2020	800	830	2909	442 K
	lockdown 1	11-03-2020	31-05-2020	81	891	3317	(approx. 0.35%)
	post-lockdown 1	01-06-20201	22-10-2020	143	1153	4662	
	lockdown 2	23-10-2020	20-05-2021	209	1327	5643	
	post-lockdown 2	21-05-2021	31-12-2021	225	1568	7035	
Germany (83.8 M)	pre-lockdown	01-01-2018	08-03-2020	798	10 866	29 294	6570 K
	lockdown 1	09-03-2020	05-05-2020	57	12 349	37 405	(approx. 0.38%)
	post-lockdown 1	06-05-2020	14-10-2020	161	17 159	68 498	
	lockdown 2	15-10-2020	09-10-2021	359	24 410	118 551	
	post-lockdown 2	10-10-2021	31-12-2021	83	25 363	125 955	
Lithuania (2.7 M)	pre-lockdown	01-01-2018	15-03-2020	805	12 573	68 505	250 K
	lockdown 1	16-03-2020	16-06-2020	92	14 032	81 381	(approx. 10%)
	post-lockdown 1	17-06-2020	06-08-2020	50	14 751	89 338	
	lockdown 2	17-08-2020	18-04-2021	254	18 140	129 740	
	post-lockdown 2	19-04-2021	31-12-2021	257	20 348	157 254	

Sonar data comprised individual daily sonar use events, identified through a unique encoded user ID, including the time and geographical coordinates of the point at which the sonar device was first used on a given fishing trip. ‘Trips’ often consisted of multiple readings at slightly different locations from the initial point of use. For each new reading, the user can select to either start a new trip or continue the same trip. We treated all trips by one user conducted within a single 24-hour period as one trip. We then used the starting coordinates of each trip to assign it to a single, country-relevant administrative unit: municipalities, in the case of Lithuania and Denmark, regions (Kraje) in the case of the Czech Republic and districts (Landkreis) in Germany. This assignment allowed us to assess the relationship between angling effort and human population sizes. To ensure that only trips to officially recognized water bodies were included in the analysis (thereby excluding minor water bodies such as backyard dams and pools), we filtered the data for each country through a spatial layer of water bodies obtained through the Georeferencinio pagrindo kadastras (GRPK) spatial dataset of (geo)reference base cadastre (open source data) from the Ministry of Agriculture of the Republic of Lithuania, the package RCzechia [44] for the Czech Republic and the Esri Deutschland Open Data Portal [45] for Germany. We applied a buffer of 100 m around each water body for this data filtering to ensure that all shore anglers were included. Data from Denmark posed two challenges that required a different approach. First, there are over 120 000 lakes (greater than 0.01 ha), many of which are not included in the Danish spatial layer of water bodies [46]. A visual inspection of the dataset revealed few (less than 10) instances of trips that were recorded away from water bodies (e.g. in buildings—presumably to test the device), so the data were not filtered by water bodies. Moreover, unlike in other countries, many device users in Denmark were sea anglers who fished offshore (25% of all recorded trips within Denmark). We included these anglers in the analysis by creating a 10 km buffer of spatial polygons along the coast based on the closest land polygon delimited by the spatial layer of municipalities in Denmark. This approach allowed us to assign each sea angling trip to the closest municipality of origin. All data processing was conducted in R (v. 4.2.1) [47], using *tidyverse* (v. 1.3.2) [48] for data manipulation and *sf* (v. 1.0.8) [49] for spatial analyses.

We collated relevant information for each country to obtain an indication of device users versus the total number of all anglers in a country, including those that fish only occasionally. In Germany, the total number of anglers in each administrative unit was estimated based on questionnaires published in Allensbach [50]; in Denmark, this estimate was based on an omnibus survey [51]; in the Czech Republic, all anglers must register with an angling club, and the numbers are available through a register maintained by the Ministry of Agriculture [52]. In Lithuania, anglers can purchase annual, monthly or one-day licences, with around 75 000, 20 000 and 140 000 of respective licences sold annually during 2018–2021 (Ministry of Environment, Lithuania). How many anglers purchase several monthly or one-day licences needs to be clarified. However, price differences were minor (14 euros for annual, 5 euros for monthly and 1.4 euros for daily), so monthly and daily licences are probably purchased by anglers who fish only occasionally. In addition, children under 16 and retirees do not require fishing licences, but fishing is popular among retirees. Given that the assessments of angler numbers in other countries include regular and occasional anglers, we used an approximate estimate of 250 000 anglers for Lithuania.

### 2.2. Spatial and temporal COVID-19 lockdowns

Government-imposed COVID-19 restrictions during the pandemic varied among and within European countries (table 1). To ensure a consistent methodology, we used the Our World in Data compilation of ‘Policy Responses to the Coronavirus Pandemic’ curated by Mathieu *et al*. [53], available at: https://ourworldindata.org/policy-responses-covid (last accessed on 17 October 2022). This compilation of restrictions was collected and standardized by the Oxford Coronavirus Government Response Tracker (OxCGRT) [54] and has been used in other similar studies on human response to the COVID-19 pandemic [9]. To define the lockdowns in our study, we used the daily index of ‘Stay-at-home requirements', with any level of stay-at-home requirement (1–3) treated as a phase of ‘lockdown’. Based on this approach, each of the four countries had a pre-lockdown phase, starting 1 January 2018 (which was the first day of the available data), followed by two lockdowns and two post-lockdown phases (table 1, electronic supplementary material, figure S1). The length of these phases differed slightly among countries, but the overall pattern was similar. We used a different approach for Denmark because Our World in Data compilation showed a protracted first lockdown (from 20 March to 20 October 2020), even though Denmark's Ministry of Health data indicates no or minimal restrictions during the summer of 2020. For this reason, we followed the methodology of Gundelund & Skov [29] and defined the first lockdown from 10 March to 31 May 2020, and the second lockdown starting 23 October 2020. The start of the second lockdown in Denmark was gradual and accelerated during autumn. Still, we chose 23 October as the starting point, because this was when the government allowed up to 10 people to assemble [55]. The end of the second lockdown followed Our World in Data compilation. However, we ignored a one-week (from 16 to 22 November 2020) pause during Denmark's second nationwide lockdown because it was probably too short to impact fishing effort. For all countries, the end of the second post-lockdown phase and the last day of our data was 31 December 2021.

### 2.3. Statistical analysis

We fit a generalized linear mixed model (GLMM) to data from each country with the phase of lockdown (pre-lockdown, during lockdown 1, post-lockdown 1, during lockdown 2, post-lockdown 2), day of the week (weekday, weekend), administrative unit population size, the number of unique sonar users, and interactions between the phase of lockdown and day of the week as fixed effects. Because fishing effort varies extensively across seasons and to ensure that lockdown effect was separated from the season effect we included season (spring, summer, autumn, winter) as a random effect. Fishing effort may also vary across years, and within a given municipality or administrative unit it will depend on the available fishing opportunities and population density. Therefore year (2018–2021) and national administrative region were also included as random effects. The number of administrative units in each country was: the Czech Republic 14, Denmark 96, Germany 374 and Lithuania 58 (electronic supplementary material, table S1). Lithuania and Denmark have more units, but some did not have any sonar device users recorded during the study and were removed from the analysis. We also accounted for the increasing uptake of this device over the studied period by including the cumulative number of unique device users as a covariate in the model. Data were initially modelled with a Poisson-distributed response, which assumed that the variance was equal to the mean. Overdispersion occurs when the observed variance in the data is higher than the expected variance from the model, and we tested the dispersion ratio of models using the *performance* package (v. 0.9.2.4) [56]. All Poisson models were overdispersed, so we assumed a negative binomial error structure instead.

The final model fitted to the data for each country took the form

$$SDU_{ijkl}∼NegBin(\mu_{ijkl,}\theta ),$$

$$E(SDU_{ijkl})=\mu_{i}\;\mathrm{and}\mathrm{var}(SDU_{ijkl})=\mu_{ijkl}+(\mu_{ijkl}^{2}/\theta ),$$

$$\log(\mu_{ijkl})=\eta_{ijkl},$$

$$\eta_{ijkl}=Day_{ijkl}+Phase_{ijkl}+Day_{ijkl}:Phase_{ijkl}+Population_{ijkl}+Unique_{ijkl}+Season_{j}+Year_{k}+AU_{l}+\varepsilon_{ijkl},$$

$$Season_{j}∼N(0,\sigma_{Season}^{2}),$$

$$Year_{k}∼N(0,\sigma_{Year}^{2}),$$

$$AU_{l}∼N(0,\sigma_{AU}^{2}),$$

$$\varepsilon_{ijkl}∼N(0,\sigma^{2}),$$

Where *SDU_ijkl_* is the number of sonar device users on day *i* in *Season j* of *Year k* in administrative unit (*AU*) *l*, assuming a negative binomial distribution with mean *μ_ijkl_* and variance *μ_ijkl_* + (*μ_ijkl_*^2^/*θ*). The parameter *θ* is the dispersion parameter. *Day* is a categorical variable for weekend/weekday and *Phase* for the phase of COVID-19 lockdown. *Day* (weekend/weekday) and *Phase* were treated as fixed effects, and we also included the interaction between these two effects to test whether COVID-19 lockdowns affected fishing effort differently during weekdays and weekend. *Population* is a continuous variable representing population density for the administrative unit. The variable *Unique* is a cumulative count of unique sonar device users on each day (electronic supplementary material, figure S2) and was included in the model to accommodate the increase in new sonar device users over time. The random intercept *Season_j_* was included in the model to introduce a correlation structure between observations for different administrative units in the same season (spring: March to May, summer: June to August, autumn: September to November, winter: December to February), with variance $\sigma_{Season}^{2}$ distributed normally and equal to 0. Similarly, *Year_k_* was included to accommodate correlation in the data between observations for different administrative units in the same season in the same year (2018–2021), and *AU_l_* for correlation between observations for the same administrative units in the same season in the same year, while *ε_ijkl_* is the residual variance in the model, with an assumption of normality and mean equal to 0.

We estimated the change in angling effort due to the lockdowns by comparing two estimates of the total effort in each phase of lockdown: an ‘observed’ estimate that used model parameters that were derived from the phase in question and a ‘null’ estimate that assumed no lockdown but other seasonal, annual, spatial and uptake effects estimated by the model from the pre-lockdown phase (table 2). All details of these calculations, aggregated data and analysis codes are available in the GitHub repository https://github.com/astaaudzi/covid_angling and at [57].

**Table 2.** Summary of fixed effects for negative binomial GLMMs fitted to sonar user data for the four countries investigated in the study. Results show the impact of phase of COVID-19 lockdown (pre-lockdown, during lockdown 1, post-lockdown 1, during lockdown 2, post-lockdown 2), day of week (weekday, weekend), regional population size, number of unique sonar users and interactions between phase of lockdown and day of week. The pre-lockdown phase and weekday are the intercept of the models. The marginal and conditional *R*^2^ indicate the proportion of variance in sonar usage data explained by the model fixed effects and fixed + random effects, respectively. Details on model random effects are shown in electronic supplementary material, figures S3–S6 and table S1.

	country											
	Czech Republic (*N* = 20 454)			Denmark (*N* = 140 256)			Germany (*N* = 546 414)			Lithuania (*N* = 78 184)		
coefficient	estimate	95% CI	*p*-value	estimate	95% CI	*p*-value	estimate	95% CI	*p*-value	estimate	95% CI	*p*-value
intercept	−0.11	−0.95 to 0.72	0.793	−3.65	−4.04 to −3.01	<0.001	−3.07	−3.51 to −2.62	<0.001	−0.83	−1.14 to −0.36	<0.001
day_(weekend)_	0.50	0.46–0.54	<0.001	0.68	0.60–0.76	<0.001	0.59	0.56–0.62	<0.001	0.97	0.95–0.99	<0.001
population size	0.35	−0.02 to 0.72	0.061	0.38	0.18–0.59	<0.001	−0.18	−0.33 to −0.02	0.024	0.10	−0.12 to 0.33	0.379
unique users	0.57	0.46–0.67	<0.001	0.46	0.24–0.69	<0.001	0.46	0.41–0.52	<0.001	−0.19	−0.22 to −0.10	<0.001
phase_(lockdown1)_	0.80	0.68–0.91	<0.001	0.20	0.03–0.38	0.023	1.00	0.93–1.06	<0.001	1.33	1.27–1.39	<0.001
phase_(post1)_	0.53	0.42–0.64	<0.001	−0.08	−0.28–0.12	0.434	1.11	1.05–1.17	<0.001	1.23	1.16–1.30	<0.001
phase_(lockdown2)_	0.28	0.15–0.40	<0.001	−0.91	−1.17 to −0.65	<0.001	0.69	0.62 to 0.76	<0.001	0.92	0.85 to 0.98	<0.001
phase_(post2)_	0.72	0.56–0.88	<0.001	−0.83	−1.16 to −0.50	<0.001	0.16	0.08–0.25	<0.001	1.03	0.95–1.12	<0.001
phase_(lockdown1)_ × day_(weekend)_	−0.17	−0.26 to −0.07	<0.001	−0.16	−0.32–0.01	0.065	−0.13	−0.18 to −0.07	<0.001	−0.36	−0.41 to −0.30	<0.001
phase_(post1)_ × day_(weekend)_	−0.12	−0.19 to −0.04	0.002	−0.14	−0.28 to −0.01	0.040	−0.22	−0.25 to −0.18	<0.001	−0.32	−0.39 to −0.26	<0.001
phase_(lockdown2)_ × day_(weekend)_	0.26	0.17–0.35	<0.001	0.26	0.10–0.41	0.001	0.00	−0.03 to 0.03	0.943	−0.04	−0.08 to 0.01	0.099
phase_(post2)_ × day_(weekend)_	−0.04	−0.10 to 0.02	0.196	−0.25	−0.39 to −0.11	<0.001	0.12	0.07–0.18	<0.001	−0.19	−0.24 to −0.16	<0.001
marginal/conditional *R*^2^	0.30 / 0.70			0.06 / 0.30			0.11 / 0.51			0.14 / 0.53		

## 3. Results

**Figure 1.**
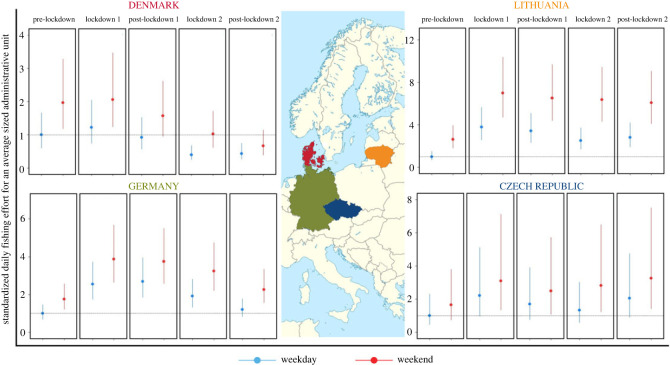
Estimated mean daily standardized fishing effort (as the number of sonar device users) per administrative unit during weekdays and weekends by country, shown for each study period (pre-lockdown, during lockdown 1, post-lockdown 1, during lockdown 2, post-lockdown 2). The effort was estimated for each country's average administrative unit size after accounting for variation due to season, year and the uptake of the device. The effort is scaled to the weekday of the pre-lockdown stage, which is therefore set to 1 (shown as a dashed horizontal line). For effort predictions across all administrative unit population sizes, see electronic supplementary material, figure S9. The error bars represent 95% confidence intervals from the general linear mixed effect model predictions. Note that visualization of single fixed effects in mixed effect models does not reveal the full effect size, since a lot of variation is explained by random and other fixed effects. Therefore, fixed effect significance should not be assessed from this figure, but from model outputs presented in table 2.

Generalized linear mixed model showed that COVID-19 lockdowns had substantial and relatively consistent effects on estimated recreational angling effort in three of four study countries. The first lockdown phase in the boreal spring of 2020 lasted between 57 (Germany) to 81 (Denmark) days and was generally associated with substantial and significant increases in the estimated effort. Effort in the Czech Republic, Germany and Lithuania increased 2.2–3.8 times (*p* < 0.001, figure 1, tables 2 and 3) and remained substantially higher (1.7–3.4 times higher than before the lockdowns) even after the first lockdown in summer and autumn of 2020. The second lockdown phase broadly corresponded with the boreal autumn and winter of 2020–2021, when fishing effort tends to be low relative to spring and summer (see electronic supplementary material, figures S3–S6 for the plots on random model effects). Random effects in the model accommodated this seasonal variation in effort, and the results show that fishing effort during the second lockdown in the Czech Republic, Germany and Lithuania was still much higher than before the lockdowns. Germany was an exception in that the second lockdown phase lasted almost a year, but fishing effort during this period remained 1.3–2.5 times higher than before the lockdowns. The increased fishing effort was sustained in all three countries even after all lockdowns ended in mid-2021 (1.2–2.8 times higher than before the lockdowns). Denmark showed a slightly different pattern than the other three countries. There was about a 20% increase in effort during March–May 2020 (*p* = 0.023), with a return to pre-lockdown effort levels during the summer of 2020 (first post-lockdown period) and strong indications of a 50–60% reduction in effort during the second lockdown and second post-lockdown phase compared with the pre-lockdown stage.

**Table 3.** Total number of predicted sonar user trips for the four countries for each lockdown period based on negative binomial GLMMs (also shown in electronic supplementary material, figure S8). Because the number of trips depends on the population size and unique users, these predictions use average population sizes and the average number of unique users in each country. Results show the predicted total number of user trips for each of the studied periods with and without the lockdown effect included and their ratio. Numbers in parentheses show 95% confidence intervals. For all calculation details, see Excel spreadsheet at https://github.com/astaaudzi/covid_angling or [57].

country	lockdown phase	number of trips	number of trips without lockdowns	ratio
Czech Republic	pre-lockdown	11 947 (5077–27 841)	—	—
	lockdown 1	2389 (875–6510)	1074 (433–2594)	2.2 (2.0–2.5)
	post-lockdown 1	3410 (1270–9126)	2007 (834–4812)	1.7 (1.5–1.9)
	lockdown 2	3763 (1346–10 355)	2844 (1159–6941)	1.3 (1.2–1.5)
	post-lockdown 2	7916 (2806–22 158)	3853 (1603–9191)	2.1 (1.8–2.4)
Denmark	pre-lockdown	2904 (1668–4991)	—	—
	lockdown 1	336 (154–742)	275 (149–507)	1.2 (1.0–1.5)
	post-lockdown 1	448 (201–991)	486 (266–879)	0.9 (0.8–1.1)
	lockdown 2	344 (141–835)	855 (454–1600)	0.4 (0.3–0.5)
	post-lockdown 2	321 (127–810)	737 (405–1336)	0.4 (0.3–0.6)
Germany	pre-lockdown	17 037 (10 837–27 060)	—	—
	lockdown 1	3103 (1812–5344)	1142 (715–1851)	2.7 (2.5–2.9)
	post-lockdown 1	9543 (5667–16 306)	3145 (1983–5061)	3.0 (2.9–3.2)
	lockdown 2	15 265 (8944–26 339)	7657 (4811–12 318)	2.0 (1.9–2.1)
	post-lockdown 2	2176 (1250–3887)	1854 (1154–3027)	1.2 (1.1–1.3)
Lithuania	pre-lockdown	29 887 (21 698–48 315)	—	—
	lockdown 1	10 807 (7259–18 983)	2858 (2039–4728)	3.8 (3.6–4.0)
	post-lockdown 1	5356 (3536–9499)	1566 (1108–2589)	3.4 (3.2–3.7)
	lockdown 2	23 228 (15 417–40 910)	9257 (6590–15 354)	2.5 (2.3–2.7)
	post-lockdown 2	24 232 (15 897–43 422)	8651 (6148–14 168)	2.8 (2.6–3.1)

As expected, recreational fishing effort was considerably higher during weekends than on weekdays. This difference probably reflected the real difference in fishing effort, but also a higher probability of device use during weekends since the device may be more popular among employed anglers with higher incomes [26]. Notably, one of the most consistent features of fishing activity during the first lockdown stage was the significant negative interaction between effort and weekend (table 2), indicating a more even distribution of fishing activities during the week (electronic supplementary material, figure S7, table 2). However, the pattern was less consistent during the second lockdown, where the difference in effort distribution between weekdays and weekends increased, rather than decreased, compared with the pre-lockdown levels in the Czech Republic and Denmark (*p* < 0.001) but not in Germany or Lithuania (table 2).

In addition to assessing changes in recreational fishing effort during the COVID-19 lockdowns, our models explained a surprisingly large proportion of variance (51–70%) through space and time in Germany, the Czech Republic and Lithuania (conditional *R*^2^ in table 2). This result suggests that the temporal and spatial distribution of device use and, by inference, recreational fishing effort was consistent and predictable. The models explained less variation for Denmark (31%), possibly due to the low uptake of the device in this country (estimated at 0.35% of total anglers, table 1). The fixed effects of lockdown phase, weekend and population density explained 11–30% of the variation in Germany, the Czech Republic and Lithuania and 6% in Denmark. The remaining variance (40% in the Czech Republic, 24% in Denmark, 40% in Germany and 39% in Lithuania) was explained by the random effects, comprising administrative unit, year and season (electronic supplementary material, figures S3–S6; table 2). Except for Germany, predicted angling effort tended to be greater in municipalities or counties with larger population sizes, although the effect was only significant in Denmark. This observation suggests that anglers were more likely to fish within a short distance of their homes rather than travel in search of fishing opportunities. By contrast, the significant negative association between population size and angling effort in Germany (*p* = 0.024, table 2) indicates that more angling occurs in less populated areas.

## 4. Discussion

Our study demonstrated substantial and broadly consistent COVID-19 lockdown-driven changes in recreational fishing effort across four European countries with different socio-economic conditions and fishing regulations. The estimated fishing effort in Lithuania, Germany, and the Czech Republic increased two- to three-fold during the first lockdown of spring 2020 (first research question) and became more evenly distributed during the week. Notably, increases in recreational fishing effort persisted until the end of 2021, almost two years after the imposition of the first lockdown (second research question). By the end of 2021, this increase remained about two times higher than the pre-lockdown period in Lithuania and the Czech Republic and approximately 10–30% higher in Germany. The persistence of elevated fishing effort hints at extensive and long-term societal changes resulting from COVID-19 lockdowns. Such changes are widely documented in economic systems [58], healthcare [59] and travel [60] but are less recognized in the context of outdoor recreation and potential human impacts on the natural world. Anecdotal evidence and published studies have already suggested that the COVID-19 lockdowns drove significant increases in recreational fishing activity, especially in the developed world [9,29–31,61]. However, none of these studies had sufficiently long time series of data to assess whether changes were sustained in the medium term. To our knowledge, ours is the first study to document recreational effort changes using daily data from across four countries for an extended period.

The two- to three-fold increase in recreational fishing effort that we observed was considerably higher than estimates from other studies, e.g. 20% increases in Denmark [29], Canada [61] and the United States [30]. Moreover, while responses across smaller spatial and temporal scales may be complex (e.g. Ryan *et al*. [32] in Western Australia), we show that when analysed on a larger scale, human behaviour changes were surprisingly consistent across countries, despite different COVID-19 regulations and recreational fishing traditions. This consistency suggests that similar changes in recreational fishing have probably occurred in other developed countries and perhaps globally. The most consistent response occurred during the first lockdown in March–May 2020, with increased effort across all four studied countries and relatively more fishing occurring during weekdays. The first lockdown was the period with the most consistent and stringent restrictions and policies. Many businesses and schools were closed, and lockdown measures were strictly enforced. Policies during the subsequent phases of post-lockdown and second lockdown were move variable, which probably translated into greater among-country variation in the intensity and weekly distribution of fishing activity. Interestingly, observed recreational effort appeared to track EU unemployment data, suggesting a potential secondary effect of COVID-19 on recreation through job losses and increased free time [62] (electronic supplementary material, figure S8).

Our estimates of recreational fishing effort in Denmark were unique in that we observed a relatively small, approximately 20% initial increase (95% CI range of 0–50%, table 3) in effort, followed by a subsequent decrease in 2021 to levels lower than before the lockdowns. The initial increase during the spring 2020 lockdown was similar in magnitude to the increase in fishing licence sales reported in Gundelund & Skov [29]. The relative increase in weekday and decrease in weekend angling effort was also consistent with what has been found among users of the Fangstjournalen digital citizen science platform [29]. It is harder to explain the apparent large decrease in the estimated fishing effort in the post-lockdown period and during 2021. The sale of the mandatory annual angling licences in Denmark was indeed lower in 2021 (151 686 licences) compared with 2020 (169 931 licences) but still well above the pre-lockdown, i.e. in 2018 (135 082 licences) and 2019 (137 133 licences) [63]. Hence, both the sonar device and licence sales data support the observation that an increase in recreational angling in 2020 was not sustained in 2021, but they paint a different picture of the decrease in effort during 2021 compared with the pre-lockdown levels. On the one hand, this discrepancy could be driven by a low uptake of sonar devices in Denmark. The absolute number of device users in Denmark is considerably lower than in other countries (table 1), which could make the data more influenced by the behaviour of a subset of active users, especially if they are responsible for a significant proportion of total trips. On the other hand, it is conceivable that the anglers who bought annual angling licences in 2021 intended to fish more than during the pre-lockdown period but ultimately went on fewer fishing trips. Resolving this discrepancy requires data for calibrating sonar device usage to the actual number of anglers in Denmark, as was done in Lithuania [42] and for the citizen science platform Fangstjournalen users in Denmark [64].

Given the difficulties of estimating recreational fishing effort directly, many studies use fishing licence sales as an indication of effort or even the only data source [30,64–66]. Yet, the discrepancy we observed between annual licence sales and the number of fishing trips estimated with the sonar device usage is not unique to Denmark. For example, the large increase in effort observed in Lithuania was not reflected in the annual angling sales during 2020–2021 (76 000 on average) compared with the previous periods (74 000 on average, during 2015–2018), and only minor changes in monthly (19 000 versus 21 000; same periods) or 2-day licence (14 000 versus 16 000) sales. Licence numbers are even harder to track in Germany, where licences are sold for different water bodies separately by various organizations and fishing clubs, and no national register exists. These observations indicate considerable difficulties in obtaining accurate estimates of recreational angling effort, given that anglers in most countries are not required to report individual fishing trips. The challenge is even greater during societal shifts when human behaviour changes quickly, and existing data collection methods are not equipped to track these changes. Our study demonstrates an alternative approach for estimating recreational effort in near real-time and at high temporal and spatial resolutions. However, our approach assumes that sonar usage reflects the recreational fishing effort, which may not be the case if sonar users behave differently from the majority of anglers, or if the usage of the device itself changed during the pandemic (e.g. anglers were more likely to use it). An important follow-up study would be to assess whether changes in fishing effort were largely driven by new users, increased number of trips by already avid anglers or both. Data could also be biased if device use is more prevalent in certain types of fishing than others, which is likely to be the case. Yet, the overall consistency of our findings with general expectations of effort change through different lockdown phases (initial increase and subsequent decrease) suggests that the biases might be reasonably small, and device usage may represent a reliable proxy of the total effort.

Our study is not the first to use novel digital data to assess human activities and recreational fishing. Smartphone applications and other digital methods, e.g. web scraping, provide an increasingly common approach to explore fishing effort and catches. These methods differ from traditional data collection techniques (e.g. creel surveys, recall surveys, boat ramp and aerial surveys), which aim for random sampling and probabilistic methods [67]. By contrast, app and other digital methods are often based on self-selection, which makes them non-probabilistic and probably biased [39]. Although probabilistic surveys generally outperform non-probabilistic methods, they can be complex and expensive and are still susceptible to bias [68–71]. Moreover, digital approaches to angler effort have often provided effort estimates similar to those of traditional survey methods. For example, Papenfuss *et al*. [72] found a positive relationship between creel-based estimates and app-reported fishing trips in Alberta, Canada. Similarly, Gundelund *et al*. [64] found a significant positive relationship between anglers counted during aerial surveys and active users of the Fangstjournalen smartphone application, which indicated that approximately 10% of the observed anglers were citizen science participants. Data from the electronic citizen science platform MyCatch provided similar patterns of regional fishing activity and spatial distribution of participants compared with traditional survey methods in Alberta, Canada [73]. Further, 40 aerial surveys of the Kaunas Reservoir in Lithuania in 2020 showed that the number of the Deeper sonar users, the device used in this study, accurately reflected total angler numbers with narrow Bayesian posterior probability ranges (95% posterior probability of 1.5–2.6% of anglers using the device during the open water season) [42]. These studies point to the potential of electronic angler platforms for providing high-resolution spatial and temporal data in near real-time, which is important and timely, given the growing popularity of recreational fishing and the lack of data for estimating recreational effort and catches in most countries [74]. However, to ensure that electronic data platforms provide reliable and widely acceptable results, we urgently need validation and calibration studies to estimate possible biases and monitor changes in device usage over time.

**Figure 2.**
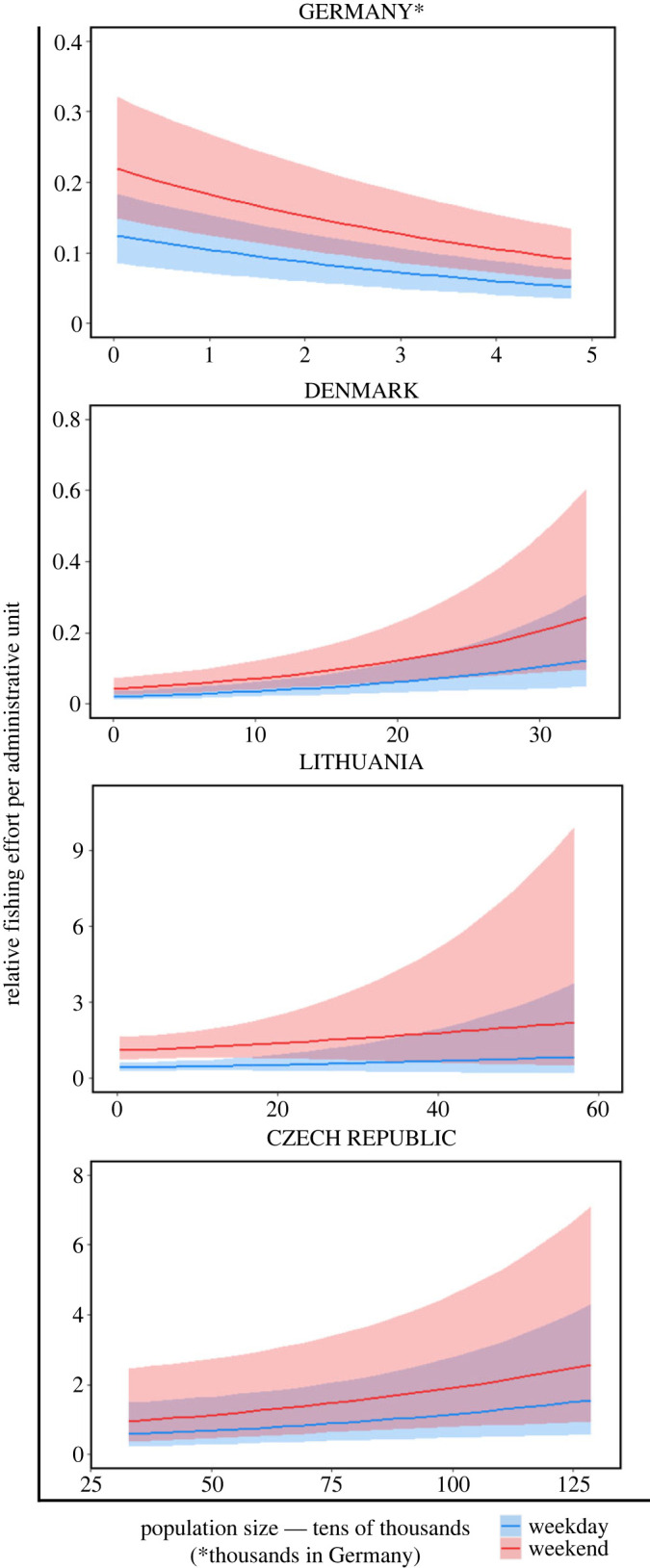
Association between relative fishing effort per administrative unit and human population density for the pre-lockdown period. Associations remained similar for all five studied periods (electronic supplementary material, figure S9).

To address our third question, regarding factors that explain the estimated angling effort through space and time, we note that statistical models applied here explained a surprisingly high proportion of variance, given the inherently noisy nature of human behavioural data (electronic supplementary material, table S1, electronic supplementary material, figures S3–S6). We also observed a positive association between estimated angling effort and the human population density in Denmark, Lithuania and Czech Republic, contrasting with a negative association in Germany. This association is evident in figure 2 and electronic supplementary material, figure S9. However, it was not identified as significant in our Lithuania and Czech Republic models because of a high correlation with the municipality random effect (see Methods, table 2). A positive association with population size might indicate that many anglers fish close to their homes and that fish populations in densely populated areas are likely to be under higher recreational fishing pressure compared with more remote locations. While this is expected in large countries like Canada and the USA (although see Weir *et al*. [75] for evidence of high angler movement rates in the USA), anglers in smaller countries, like Lithuania or Czech Republic, could in theory travel large distances to visit the most popular fishing locations. Yet, our data indicates that even in these countries more anglers (including avid anglers who have the sonar device) fish closer to their homes. The negative association between population size and angling effort in Germany was unexpected. It could have been driven by greater industrialization in Germany, where densely populated areas may have fewer attractive fishing opportunities. Given that the administrative units in Germany are relatively small, many urban anglers probably travel to neighbouring, less developed areas for angling [76]. This relocation of angling effort is easier in parts of the country with large angler associations, as agreements often allow anglers from nearby states to purchase discounted permits for association waters. The decreasing acceptance of angling as a recreational activity [77] may also drive anglers to rural areas where potential confrontations may be less likely.

In summary, our study suggests an approximate doubling of recreational fishing effort across Europe in response to the COVID-19 lockdowns, with concomitant important societal, economic and ecological consequences. On the one hand, recreational fishing has multiple human well-being and economic benefits, which have potentially provided valuable positive contributions to local economies and societies during the challenging years of the pandemic. On the other hand, such increases in effort are likely to represent a substantial increase in fishing pressure on inland and coastal ecosystems and species, many of which are already strongly affected by climate change and pollution [78–81]. While increases in fishing effort of this magnitude in marine fisheries are not uncommon, this change typically occurs over decades rather than days and weeks [82]. The implications of such acute changes in fishing effort are difficult to predict, but the short period over which these increases in effort occurred suggest that existing regulations may have been insufficient at maintaining fishing mortality within sustainable limits. In this study we assumed that recreational fishing effort is largely driven by socio-economic processes and the feedbacks with potentially declining population status did not exist or were minimal. Understanding potential impacts of increased recreational fishing pressure on inland and coastal fish populations represents an important and urgent follow-up study. However, in many cases, the inland and coastal populations targeted by anglers are unassessed [43,83], so the potential impacts may remain unknown. Closer collaboration between recreational anglers, smartphone application companies and scientists could significantly improve this situation, as digital technologies provide fast and effective ways to collect data on fish population status.

## Data Availability

The raw data were generated by users of the Deeper platform. Anonymous data were obtained through a data-sharing agreement. Requests for raw data should be directed to Vincentas Vienožinskis at Deeper (vincentas.vienozinskis@deeper.eu). Derived data and relevant code for all analyses are stored in GitHub: https://github.com/astaaudzi/covid_angling and have been archived within the Zenodo repository: https://zenodo.org/record/8072917 [84]. The data are provided in electronic supplementary material [85].
